# Short‐Term Oral Administration of the Porcupine Inhibitor, Wnt‐c59, Improves the Structural and Functional Features of Experimental HFpEF

**DOI:** 10.1002/prp2.70054

**Published:** 2025-01-01

**Authors:** Mhairi A. Paul, Cherry L. Wainwright, Emma E. Hector, Erik Ryberg, Stephen J. Leslie, Sarah K. Walsh

**Affiliations:** ^1^ School of Pharmacy and Life Sciences, Robert Gordon University Aberdeen UK; ^2^ Bioscience Cardiovascular, Research and Early Development, Cardiovascular, Renal and Metabolism (CVRM), BioPharmaceuticals R&D AstraZeneca Mölndal Sweden; ^3^ Raigmore Hospital Inverness UK

**Keywords:** cardiac function, fibrosis, HFpEF, hypertrophy, Wnt signaling, Wnt‐c59

## Abstract

Heart failure with preserved ejection fraction (HFpEF) accounts for approximately 50% of heart failure cases globally, and this incidence is increasing due to extended lifespans and accumulating comorbidities. Emerging evidence suggests that Wnt signaling plays a role in cardiomyocyte hypertrophy and cardiac fibrosis, which are key features of HFpEF. Furthermore, Porcupine (PORCN) inhibitors, which negatively regulate Wnt signaling, have shown promising results in improving cardiac function and reducing cardiac hypertrophy and/or fibrosis. This study investigated whether acute oral administration of the PORCN inhibitor, Wnt‐c59, alters the maladaptive structural and/or functional features in a mouse model of HFpEF. Male mice were given a high‐fat diet and L‐NAME (0.5 g L^−1^) in drinking water for 5 weeks, followed by a 2‐week intervention of orally administered Wnt‐c59 (5 mg kg^−1^ day^−1^). HFpEF mice were characterized by hypertension, cardiac hypertrophy and fibrosis, and diastolic dysfunction, although there was no evidence of activation of Wnt signaling in the heart. Despite this, short‐term treatment of HFpEF mice with Wnt‐c59 ameliorated adverse cardiac remodeling by increasing the ratio of the more compliant collagen type 3 to that of the more tensile collagen type 1 in the heart. Furthermore, Wnt‐c59 also improved diastolic dysfunction, which was associated with the increased cardiac expression of brain natriuretic peptide, a known promoter of ventricular compliance. Our findings demonstrate that even short‐term administration of a PORCN inhibitor improves both the structural and functional features of experimental HFpEF.

## Graphical abstract

The Porcupine (PORCN) inhibitor, Wnt‐c59, improves diastolic function in experimental heart failure with preserved ejection fraction (HFpEF).

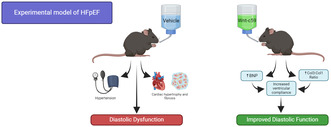

**Abbreviations**
AngII: angiotensin II
BNP: brain natriuretic peptide
BSU: Biological Services Unit
CCDN1: cyclin D1
cGMP: cyclic guanosine monophosphate
c‐Myc: cellular Myc
Col1: collagen type 1
Col3: collagen type 3
DBP: diastolic blood pressure
DMSO: dimethyl sulfoxide
*E*_a_
: arterial elastance
EDP: end diastolic pressure
EDV: end diastolic volume
EDPVR: end diastolic pressure volume relationship
EF: ejection fraction
ESP: end systolic pressure
ESV: end systolic volume
ESPVR: end systolic pressure volume relationship
HFpEF: heart failure with preserved ejection fraction
HFD: high fat diet
HW/TL: heart weight to tibia length ratio
L‐NAME: *N*_ω_‐nitro‐L‐arginine methyl ester hydrochloride
LV: left ventricle
NO: nitric oxide
PKG: protein kinase G
PORCN: Porcupine
PVL: pressure volume loop
PSR: picrosirius red
RAAS: renin‐angiotensin‐aldosterone system
SBP: systolic blood pressure
SV: stroke volume
Vp: parallel conductance
Wnt: Wingless/int1

## 1 Introduction

Heart failure with preserved ejection fraction (HFpEF) accounts for approximately 50% of all heart failure cases [1], and it is predicted to rise further due to increasing life expectancy and the elevated incidences of obesity, hypertension, Type 2 diabetes mellitus, and atrial fibrillation [2, 3]. Due to a limited number of effective treatments and the increasing prevalence of comorbidities, survival rates are failing to improve, and new therapies are required. HFpEF is characterized by diastolic dysfunction, or impaired relaxation, which is due to the presence of increased ventricular stiffness [4, 5]. This reduced ventricular compliance occurs as a consequence of several different pathophysiological mechanisms, which tend to culminate in the adverse remodeling of the heart and specifically the development of both cardiomyocyte hypertrophy and cardiac fibrosis [6]. Furthermore, the renin‐angiotensin‐aldosterone system (RAAS) has been suggested as a prominent contributor to HFpEF as it is activated in this condition, and angiotensin II (AngII), its main effector peptide, has been shown extensively to promote both cardiomyocyte hypertrophy and cardiac fibrosis [7, 8, 9, 10]. Therefore, interventions aimed at modulating this system and/or its downstream mediators have the potential to prove beneficial in the setting of HFpEF.

Wingless/int1 (Wnt) signaling plays an essential role in the regulation of development, growth, and homeostatic processes [11], and while it is normally quiescent in the adult heart, it is known to be reactivated in cardiovascular disease [12]. Chronic infusion of AngII has been shown to activate both canonical (i.e., β‐catenin dependent) and non‐canonical Wnt signaling in the hearts of rodents, and canonical Wnt signaling, in particular, has been shown to contribute to the development of both cardiomyocyte hypertrophy and cardiac fibrosis [13]. Elevated β‐catenin levels have been detected in both cardiomyocytes exposed to hypertrophic stimuli [14] and hearts exposed to pressure overload [15, 16], while mice with cardiomyocyte‐specific depletion of β‐catenin have substantially reduced hypertrophy and improved cardiac function [17]. In relation to fibrosis, loss of β‐catenin function in myocardial fibroblasts and the administration of ICG 001 have both been shown to attenuate cardiac fibrosis and improve cardiac function in mice subjected to transverse aortic constriction‐induced pressure overload [18].

Since Wnt signaling appears to be reactivated in both cardiac hypertrophy and fibrosis, research has focused on Wnt inhibitors as a potential therapy. Recently, Porcupine (PORCN) inhibitors have produced promising results in experimental models of cancer [19, 20] and cardiovascular disease [21, 22, 23]. PORCN inhibitors abrogate the palmitoylation of Wnt proteins, preventing their secretion and subsequent activation of Wnt signaling pathways. Recently, chronic (i.e., 10 weeks) intraperitoneal administration of the novel PORCN inhibitor, CGX1321, was shown to attenuate both cardiac hypertrophy and fibrosis and improve cardiac function in an experimental “two hit” (both metabolic and mechanical stress) model of HFpEF via the mitigation of both canonical and non‐canonical Wnt signaling [22], demonstrating that the targeting of these signaling pathways represents a viable therapeutic target for HFpEF. A separate study has demonstrated the therapeutic potential of Wnt‐c59, a PORCN inhibitor with similar potency to CGX1321, when administered orally over a short intervention period (i.e., 2 weeks) in a mouse model of mammary cancer [20]. In that study, oral administration of Wnt‐c59 was shown to inhibit PORCN activity at nanomolar concentrations, display good bioavailability (i.e., plasma concentrations maintained above the IC_50_ (i.e., 74 pM) for Wnt‐c59), and attenuate the progression of mammary tumors in mice via the downregulation of Wnt target genes [20]. Therefore, the purpose of this study was to investigate whether acute (i.e., 2 weeks) oral administration of a similarly highly potent PORCN inhibitor, Wnt‐c59, could improve structural and/or functional features of HFpEF using the same “two hit” experimental model of the condition.

## 2 Materials and Methods

### 2.1 Animal Housing and Ethical Approval

Animal studies were conducted under an appropriate Project License in accordance with the UK Animals (Scientific Procedures) Act 1986. Male C57Bl/6J mice (8 weeks old) were purchased from Charles River (Edinburgh) and housed within the Biological Services Unit (BSU) at Robert Gordon University in a temperature and humidity (19°–23°C and 45%–65%, respectively) controlled room set on a 12 h light/dark cycle (7 am–7 pm) and maintained in accordance with the husbandry guidelines set by the UK Home Office. Only male mice were used for experimental studies, as published data available at the time of this study demonstrated that the female sex was protective in this model of HFpEF [24]. Prior to the commencement of any experiments, mice were allocated 7 days for acclimatization within the BSU, with all dietary interventions and surgical procedures conducted within the BSU. The welfare of all animals was assessed by trained staff prior to undergoing in vivo experimentation, and none were required to be excluded. All in vivo work is reported in accordance with the ARRIVE guidelines [25].

### 2.2 In Vivo Experimental Protocols

Male C57Bl/6J mice (9 weeks old; 24–24.3 g) were assigned to one of three experimental groups: (1) control; (2) HFpEF plus vehicle; and (3) HFpEF plus Wnt‐c59. The control animals were given a standard diet (cat# D12450J; Research Diets, NJ, USA) containing 10 kcal% fat while the HFpEF animals were given a high‐fat diet (HFD; cat# D12492; Research Diets, NJ, USA) containing 60 kcal% fat and Nω‐Nitro‐L‐arginine methyl ester hydrochloride (L‐NAME; 0.5 g L^−1^; cat# 15468436; Fisher Scientific, Loughborough, UK) in the drinking water for 7 weeks. Furthermore, for the final 2 weeks of the dietary/pharmacological intervention period, HFpEF mice were administered either vehicle (0.1% dimethyl sulfoxide (DMSO); cat# 10103483; Fisher Scientific, Loughborough, UK) or Wnt‐c59 (5 mg kg^−1^ day^−1^; cat# 1243243‐89‐1; Cayman Chemical, MI, USA) in drinking water containing L‐NAME (changed daily). The concentration and route of administration of Wnt‐c59 used in this study were based on previously published findings demonstrating that administration of Wnt‐c59 at this concentration in this manner achieved plasma concentrations that were above the IC_50_ (i.e., 74 pM) for this inhibitor at the PORCN enzyme in mice [20]. Original experimental group sizes were chosen on the basis of previously published literature using the same experimental model of HFpEF and measuring similar endpoints [24, 26]. However, n numbers for some functional measurements are only *n* = 3 due to technical issues with recording.

### 2.3 Measurement of Ventricular Function

Mice were weighed and anesthetized with a mixture of ketamine (120 mg kg^−1^; Narketan, Vetoquinol UK Ltd., Northamptonshire, UK) and xylazine (16 mg kg^−1^; Rompun, Bayer, Dublin, Ireland) via intraperitoneal (i.p.) injection, and the trachea cannulated to allow artificial respiration when required. To ensure core body temperature was maintained between 37° and 38°C, a heat pad (RightTemp mouse body temperature monitor; Kent Scientific Corporation, Connecticut, USA) was used. Ventricular function was measured in open‐chest mice via pressure volume loop (PVL) analysis using a method adapted from Pacher et al. [27]. Briefly, the neck was opened, the jugular vein cannulated with flame‐stretched Portex polythene tubing (0.58 mm ID × 0.96 mm OD; Smiths Medical International Ltd., Hyde, Kent, UK), and a 1.4‐Fr pressure conductance catheter (SPR‐839; Millar Inc., Houston, TX, USA) inserted into the right carotid artery. Following the measurement of arterial pressure (systolic (SBP) and diastolic (DBP) blood pressure), the catheter was removed and the artery tied. The chest was opened, and mice were artificially ventilated via a tracheostomy using the Rovent Jr. ventilation system (Kent Scientific Corporation, Connecticut, USA), and the catheter was inserted via the apex into the left ventricle. Cardiac function was then recorded via the MPVS‐Ultra Single Segment Foundation System (Millar Inc., Houston, TX, USA). To obtain measurements of load‐independent cardiac function (i.e., end‐systolic pressure‐volume relationship (ESPVR) and end‐diastolic pressure‐volume relationship (EDPVR)), venous return (i.e., preload) was varied via transient occlusion of the inferior vena cava. Finally, to enable correction of pressure volume loops during data analysis, the parallel conductance (Vp) was calculated via the administration of a small volume of hypertonic saline (15%; i.v.) to mice. Anesthesia was maintained throughout by administration of 50 μL per 25 g (b.w.) of the ketamine and xylazine mixture via i.p. injection every 40 min or as required. Following completion of the in vivo protocol, animals were euthanized via an overdose of anesthetic, blood was collected into heparinized tubes, and tissues were removed for further analysis.

### 2.4 RNA Isolation and Quantitative PCR (RT‐qPCR) Analysis

RNA was extracted from ventricular tissue (approximately 30 mg) using TRI Reagent Solution (cat# 11312940; Fisher Scientific, Loughborough, UK) following the manufacturer's guidelines. RNA concentration and purity were measured using UV absorbance spectrophotometry, with an adequate sample ratio of A260/A280 being ≥ 1.8. For the preparation of cDNA, 1 μg RNA was reverse‐transcribed using the High‐Capacity cDNA Reverse Transcription Kit (cat# 10400745; Applied Biosystems, Warrington, UK) according to the manufacturer's instructions. Briefly, each reaction consisted of 2 μL of 10X RT Buffer, 0.8 μL of 25X dNTP Mix, 2 μL of 10X Random Primers, 1 μL of MultiScribe Reverse Transcriptase, 1 μL of RNase Inhibitor, 3.2 μL DNase/RNase free water, and 1 μg of RNA, in a final volume of 20 μL. Reactions were performed on an Applied Biosystems 7900HT Fast Real‐Time PCR system (Applied Biosystems, Warrington, UK) as follows: 25°C for 10 min, 37°C for 120 min, 85°C for 5 min, and then cooled to 4°C. The cDNA was diluted with DNase/RNase free water to obtain the equivalent of 50 ng μL^−1^ of starting RNA and stored at −20°C. qRT‐PCR was performed with either a QuantiTect SYBR Green PCR kit (cat# 204143; Qiagen, Hilden, Germany) for QuantiTect primers or a SYBR Green PCR Master Mix Kit (cat# Z‐PPLUS‐R‐SY; Primer Design, Chandler's Ford, UK) for Sigma primers. Gapdh was amplified as an internal control. Primers from Sigma Aldrich were designed using the NCBI primer blast tool https://www.ncbi.nlm.nih.gov/tools/primer‐blast/ and had the following sequences: Axin2, forward 5′‐TCAGTAACAGCCCAAGAACCG‐3′, reverse 5′‐CCTCCTCTCTTTTACAGCAAAGC‐3′; cyclin D1 (CCND1), forward 5′‐TCAAGTGTGACCCGGACTGC‐3′, reverse 5′‐CCTTGGGGTCGACGTTCTG‐3′; cellular Myc (c‐Myc), forward 5′‐CCCATTACAAAGCCGCCGAC‐3′, reverse 5′‐CACTTTCGTCAGCGTGTCCA‐3′; collagen type 1 (Col1), forward 5′‐CCCTGGTCCCTCTGGAAATG‐3′, reverse 5′‐GGACCTTTGCCCCCTTCTTT‐3′; collagen type 3 (Col3), forward 5′‐TGACTGTCCCACGTAAGCAC‐3′, reverse 5′‐GAGGGCCATAGCTGAACTGA‐3′. Catalogue numbers for QuantiTect primers (Qiagen, Hilden, Germany) used are as follows: QT01658692 (Gapdh); QT00107541 (brain natriuretic peptide; BNP); QT00250439 (Wnt3a); and QT00164500 (Wnt5a). QuantiTect SYBR green qRT‐PCR amplifications were performed with an initial denaturation step at 95°C for 15 min followed by 40 cycles of denaturation at 94°C for 30 s, annealing at 55°C for 30 s, and elongation at 72°C for 30 s. Primer Design SYBR green amplifications were performed with an initial hot start at 95°C for 15 min, followed by 40 cycles of 95°C for 30 s and annealing at 60°C for 2 min. The ratio of target mRNA to Gapdh mRNA was calculated using a mathematical model previously described [28]. A negative control (DNase/RNase free water) was included for each qRT‐PCR run. Measurements of gene expression were performed in duplicate for each RNA sample, and a mean value was used for analysis. The MIQE (Minimum Information for Publication of Quantitative Real‐Time PCR Experiments) guidelines were followed [29].

### 2.5 Measurement of Wnt3a Protein Expression in Murine Ventricular Tissues via ELISA

Ventricular tissue was homogenized in a buffer (1 mL per 100 mg of tissue) consisting of cell lysis buffer (cat# 9803S; Cell Signaling Technology, Leiden, The Netherlands), a protease inhibitor cocktail (cat# P8340; Sigma‐Aldrich, Dorset, UK), and phenylmethylsulfonyl fluoride (PMSF; cat# 10485015; Fisher Scientific, Loughborough, UK). Following centrifugation (14 000×*g* at 4°C for 10 mins) protein concentration was quantified in the supernatant via the Bradford assay previously described [30]. Wnt3a protein was quantified using the Wnt3a DuoSet ELISA kit (cat# DY1324B; R&D Systems, Abingdon, UK) as per the manufacturer's instructions.

### 2.6 Extraction of Nuclear and Cytoplasmic Protein Fractions From Murine Ventricular Tissue and Measurement of β‐Catenin Protein Expression via ELISA

Nuclear and cytoplasmic protein fractions were extracted from ventricular tissue using the NE‐PER Nuclear and Cytoplasmic Extraction Reagents Kit (cat# 78833; Fisher Scientific, Loughborough, UK) as per the manufacturer's instructions. In brief, 30–40 mg of ventricular tissue was homogenized in CER I (1 mL per 100 mg of tissue), the samples incubated for 10 min on ice and then ice cold CER II (55 μL per 100 mg of tissue) added to samples. Following centrifugation (14 000×*g* at 4°C for 5 mins), the supernatant containing the cytoplasmic protein fraction was placed in a clean pre‐chilled tube. The remaining pellet was resuspended in NER (500 μL per 100 mg of tissue), samples kept on ice and vortexed every 10 min for 40 min. Following centrifugation (14 000×*g* at 4°C for 10 min), the supernatant containing the nuclear protein fraction was placed in a clean pre‐chilled tube. Protein concentration was quantified in all samples via the Bradford assay and total (both phosphorylated and unphosphorylated) β‐catenin protein quantified using the Total β‐catenin DuoSet IC ELISA kit (cat# DYC1329; R&D Systems, Abingdon, UK) as per the manufacturer's instructions.

### 2.7 Quantification of Cardiac Collagen Deposition

For Picrosirius red (PSR) staining, ventricular tissue was embedded in Optimal Cutting Temperature, and transverse sections (6 μm thickness) were cut and mounted onto glass slides. Tissue sections were fixed (buffered zinc formalin for 15 min), stained with PSR, and imaged at a magnification of ×25 using a Canon EOS 110D camera attached to a Leica DMIL microscope (Leica Biosystems, Newcastle, UK). To quantify collagen deposition, images were blinded, and the percentage area of collagen staining was calculated using computerized planimetry (ImageJ software, National Institute of Health (NIH), Rockville Pike Bethesda, MD).

### 2.8 Effects of Wnt‐c59 on the Expression of Hypertrophic and Fibrotic Markers in Fibroblasts

The mouse NIH 3T3 fibroblast cell line (cat# 93061524) was purchased from Sigma Aldrich (Dorset, UK) and cultured in Gibco Dulbecco's Modified Eagle Medium (DMEM) containing high glucose (4.5 g/L), phenol red, and sodium pyruvate and supplemented with 10% fetal bovine serum (FBS), 100 U/mL penicillin, 100μg/mL streptomycin, and 2 mM L‐glutamine. Cells were used between passages 3 and 9. NIH 3T3 fibroblasts were seeded in 6 well plates (50 000 cells per well) and treated with Wnt‐c59 (10 nM and 1 μM) for 48 h and the expression of genes related to hypertrophy (BNP) and fibrosis (Col1 and Col3) measured in cell lysates as described in section 2.4.

### 2.9 Solutions and Chemicals

All chemicals were purchased from either Sigma‐Aldrich (Dorset, UK) or Fisher Scientific UK Ltd. (Loughborough, UK) unless otherwise stated.

### 2.10 Statistical Analysis

Statistical analysis was performed using GraphPad Prism 8 software. Parametric data from three or more groups was analyzed using either a one‐way or two‐way ANOVA followed by a Bonferroni post hoc test. All data was expressed as the mean ± SEM, and results were considered statistically significant for *p* ≤ 0.05.

### 2.11 Nomenclature of Targets and Ligands

Key protein targets and ligands in this article are hyperlinked to corresponding entries in http://www.guidetopharmacology.org, the common portal for data from the IUPHAR/BPS Guide to PHARMACOLOGY [31], and are permanently archived in the Concise Guide to PHARMACOLOGY 2023/24: Enzymes [32].

## 3 Results

### 3.1 Wnt‐c59 Did Not Affect Either Obesity or Hypertension in HFpEF Mice

**FIGURE 1:**
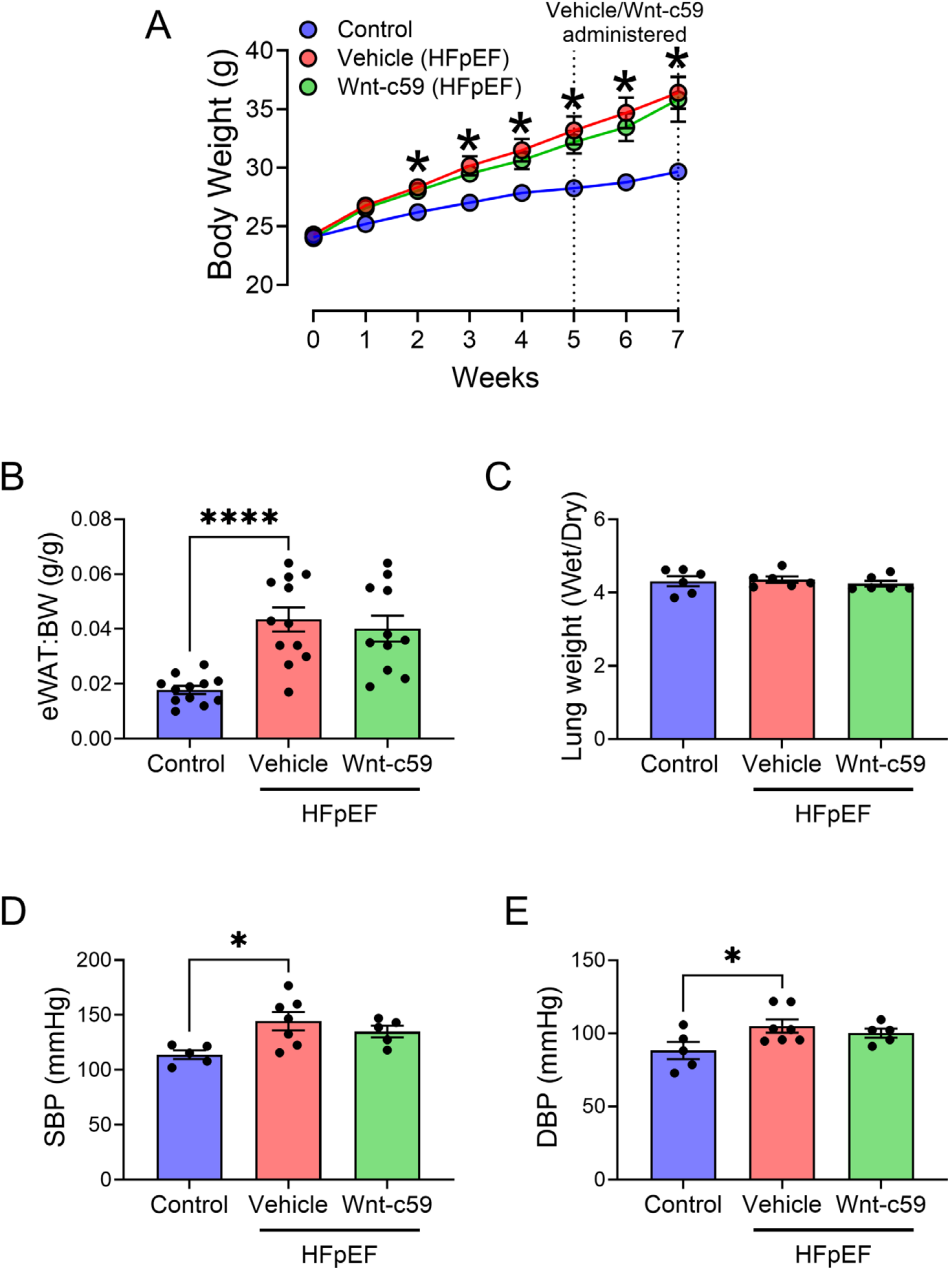
Administration of the PORCN inhibitor, Wnt‐c59, to HFpEF mice did not alter hypertension or obesity. Body weight (BW) of male C57BL/6J mice during dietary/pharmacological intervention (A), epididymal white adipose tissue weight normalized to BW (eWAT:BW) (B), ratio of wet to dry lung weight (wet/dry) (C), SBP (D), and DBP (E). Data was expressed as mean ± SEM. *p < 0.05; ****p < 0.001. *n* = 5–12.

Mice in all experimental groups had a similar starting body weight as shown in Figure 1A. In response to 7 weeks of a HFD and L‐NAME administration, HFpEF mice in the vehicle group displayed significantly increased body weight (Figure 1A; *p* < 0.05), adiposity (Figure 1B; *p* < 0.001), SBP (Figure 1D; *p* < 0.05), and DBP (Figure 1E; *p* < 0.05). However, the administration of Wnt‐c59 for 2 weeks did not significantly affect the increases in body weight (Figure 1A), adiposity (Figure 1B), or blood pressure (SBP (Figure 1D) and DBP (Figure 1E)) observed in HFpEF mice. Furthermore, there were no detectable differences in the ratio of wet to dry lung weights (Figure 1C) between any of the experimental groups.

### 3.2 Wnt‐c59 Attenuated Diastolic Dysfunction in HFpEF Mice

**FIGURE 2:**
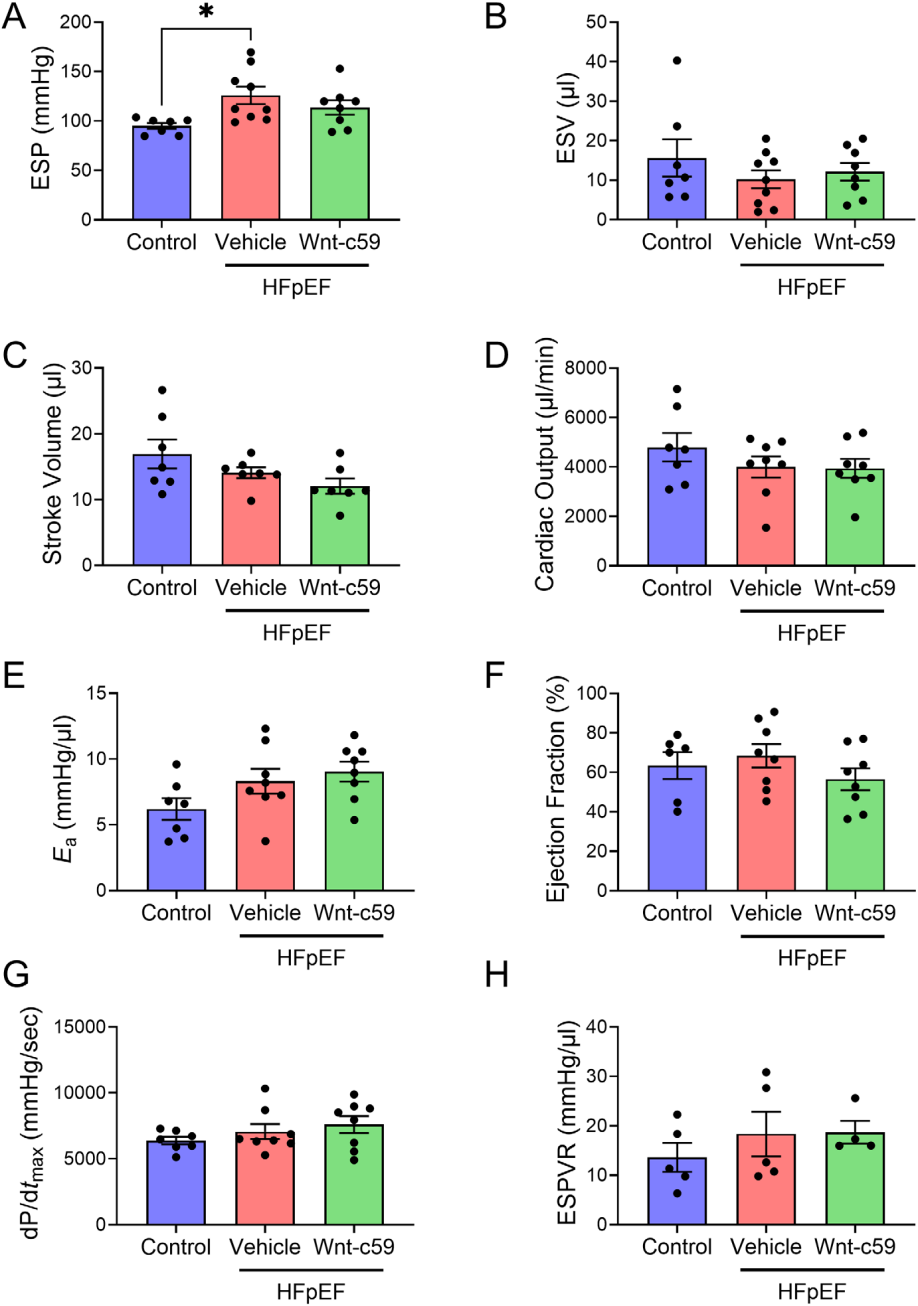
HFpEF mice did not exhibit any signs of systolic dysfunction but were characterized by elevated ESP, an affect not altered by treatment with Wnt‐c59. ESP (A), end systolic volume (ESV) (B), stroke volume (SV) (C), cardiac output (CO) (D), arterial elastance (*E*_a_) (E), ejection fraction (EF) (F), dP/d*t*_max_ (G), and ESPVR (H). Data was expressed as mean ± SEM. *p < 0.05. *n* = 4–9.

**FIGURE 3:**
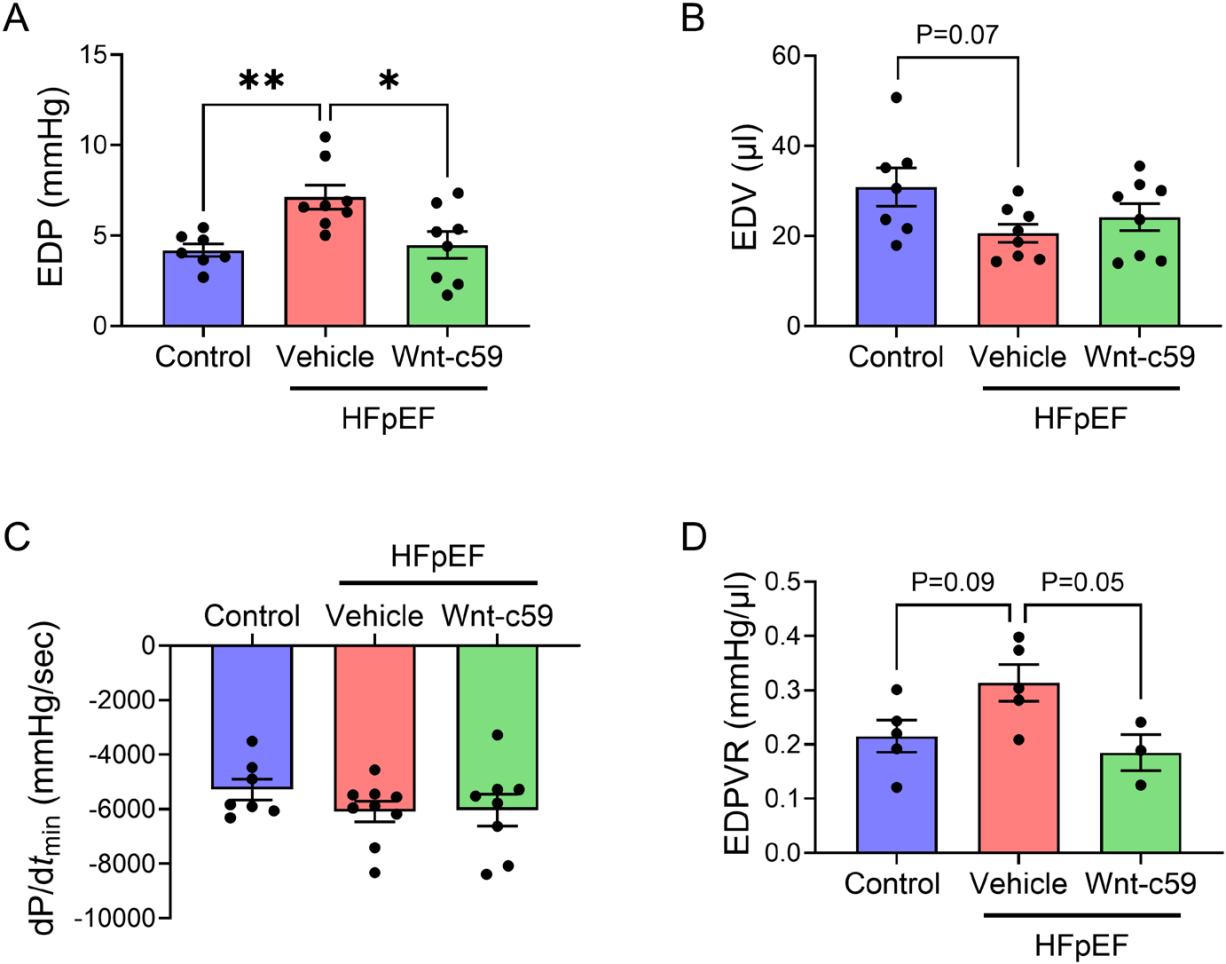
HFpEF mice exhibited signs of diastolic dysfunction including elevated EDP and EDPVR which were attenuated following treatment with Wnt‐c59. EDP (A), EDV (B), dP/d*t*_min_ (C), and EDPVR (D). Data was expressed as mean ± SEM. *p < 0.05; **p < 0.01. *n* = 3–9.

To determine if Wnt‐c59 had any effect on cardiac function PVL analysis was performed. As expected for an experimental model of HFpEF, administration of a HFD and L‐NAME to mice resulted in elevated end systolic pressure (ESP; *p* < 0.05; Figure 2A) but did not significantly affect the majority of indices of systolic function in vehicle treated mice (Figure 2B–H). Furthermore, the administration of Wnt‐c59 did not significantly affect any of the systolic parameters measured in HFpEF mice including ESP (Figure 2A–H). Conversely, indices of diastolic function were altered in HFpEF mice. In particular, end diastolic pressure (EDP) was significantly increased in the vehicle treated HFpEF group compared to control mice (*p* < 0.01; Figure 3A), and this elevated EDP was reduced in response to treatment with Wnt‐c59 (*p* < 0.05; Figure 3A). In contrast, while there was a modest reduction in end diastolic volume (EDV) in the vehicle treated HFpEF group compared to control mice, this effect was not statistically significant (*p* = 0.07; Figure 3B), indicating that this measure of diastolic function was not altered by high fat feeding and the administration of L‐NAME in mice (Figure 3B). Finally, while neither dP/d*t*_min_ (Figure 3C) nor EDPVR, a load‐independent measurement of diastolic function (Figure 3D), were significantly altered in vehicle treated HFpEF mice, HFpEF mice treated with Wnt‐c59 exhibited an EDPVR value similar to that observed in control mice (Figure 3D).

### 3.3 Wnt‐c59 Enhanced the Myocardial Expression of BNP but Did Not Affect Cardiac Hypertrophy in HFpEF Mice

**FIGURE 4:**
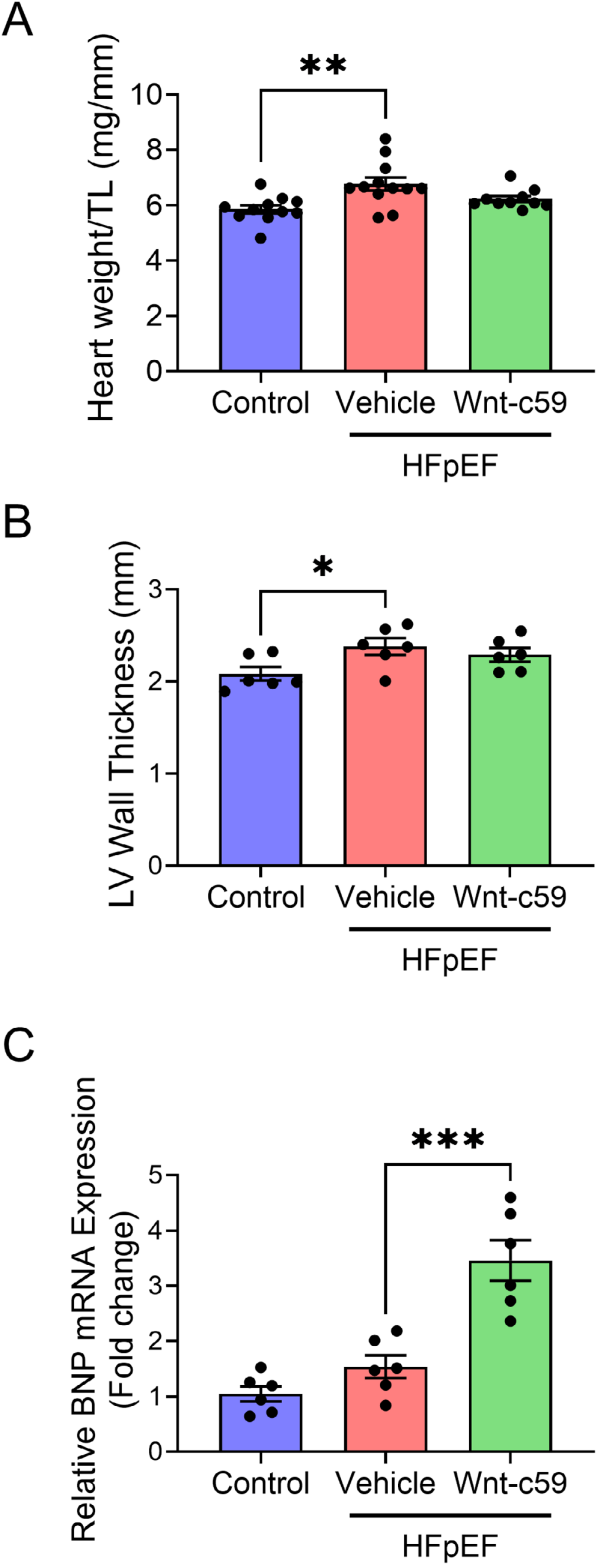
Wnt‐c59 enhanced the myocardial expression of BNP but did not affect cardiac hypertrophy in HFpEF mice. HW/TL (A), LV wall thickness (B), and mRNA expression of BNP in the heart (C). Data was expressed as mean ± SEM. *p < 0.05; **p < 0.01; ***p < 0.001. *n* = 6–12.

Vehicle treated HFpEF mice were characterized by cardiac hypertrophy, as demonstrated by a significant increase in the ratio of heart weight to tibia length (HW/TL) (*p* < 0.01; Figure 4A) and left ventricular (LV) wall thickness (*p* < 0.05; Figure 4B). While treatment with Wnt‐c59 did not significantly alter either the increased HW:TL (Figure 4A) or increased LV wall thickness (Figure 4B), it did further increase the ventricular expression of BNP compared to vehicle treated HFpEF mice (*p* < 0.001; Figure 4C).

### 3.4 Wnt‐c59 Treatment Altered Cardiac Fibrosis in HFpEF Mice

**FIGURE 5:**
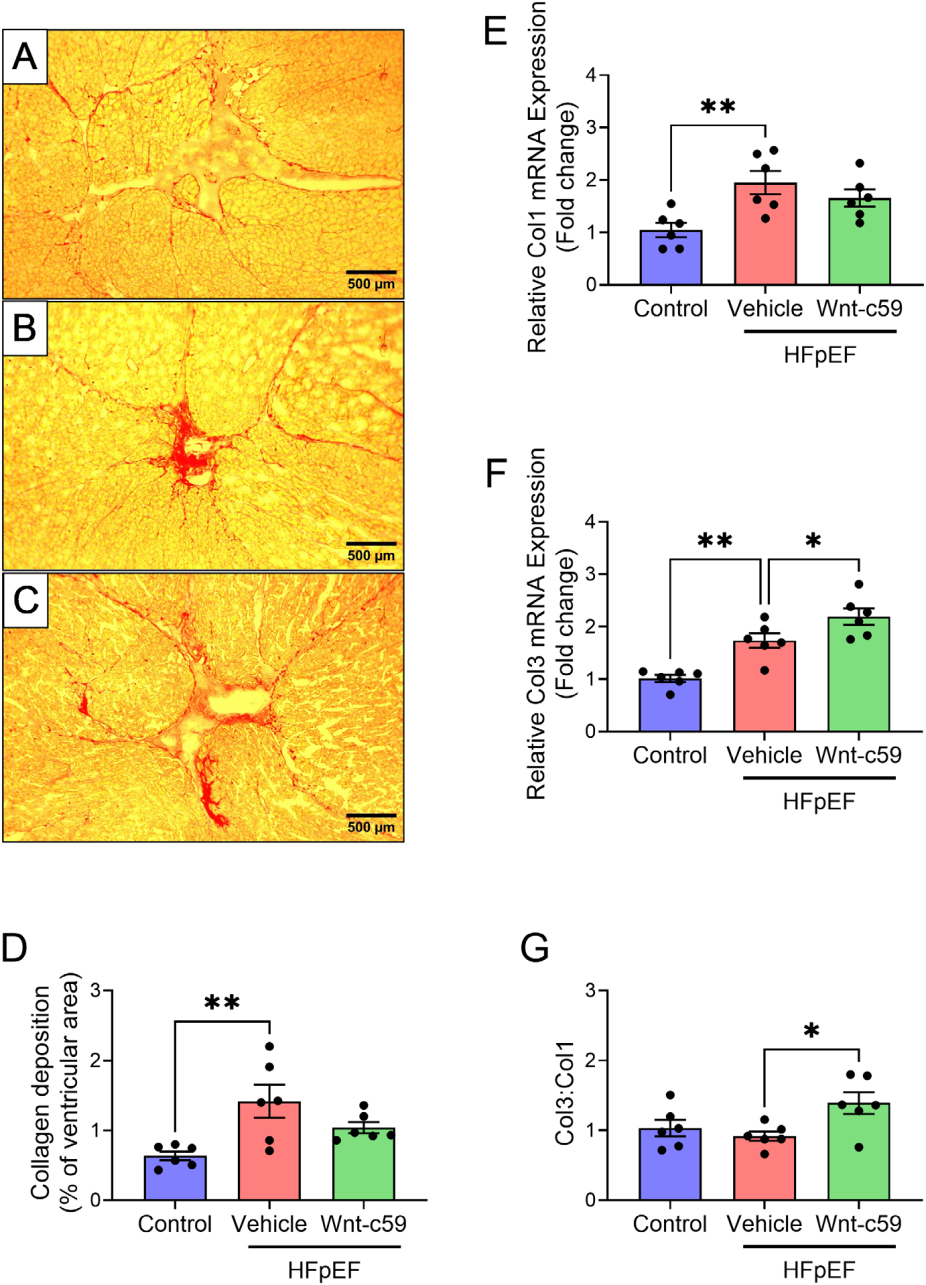
Wnt‐c59 modulated the composition of collagen subtypes in the fibrotic hearts of HFpEF mice. Representative images of PSR‐stained ventricular tissue (collagen stained red) from all experimental groups, control (A), vehicle treated HFpEF (B), and Wnt‐c59 treated HFpEF (C). Images were taken at ×100 magnification. Scale bar = 500 μm. Collagen deposition as a percentage of ventricular area (D), mRNA expression of Col1 (E), and Col3 (F) in the heart, and the ratio of Col3 to Col1 (Col3:Col1) mRNA expression (G). Data was expressed as mean ± SEM. *p < 0.05; **p < 0.01. *n* = 6.

To detect the presence of fibrosis, the mRNA expression of both Col1 and Col3 was measured in hearts in addition to the quantification of collagen deposition. PSR staining demonstrated a significant increase in the deposition of collagen in the ventricles of vehicle treated HFpEF mice (*p* < 0.01; Figure 5D). Furthermore, the mRNA expression of both Col1 (*p* < 0.01; Figure 5E) and Col3 (*p* < 0.01; Figure 5F) was also significantly increased in the ventricles of these mice, demonstrating the presence of cardiac fibrosis in these animals. While treatment with Wnt‐c59 did not significantly alter either the degree of collagen deposition (detected via histological staining; Figure 5D) or the mRNA expression of Col1 (Figure 5E) in the hearts of HFpEF mice, it did significantly increase both the mRNA expression of the thinner and more elastic Col3 (*p* < 0.05; Figure 5F) and the ratio of Col3 to Col1 mRNA (Col3:Col1) in these animals (*p* < 0.05; Figure 5G).

### 3.5 Canonical Wnt Signaling Was Not Activated in the Hearts of HFpEF Mice but Was Reduced by Wnt‐c59

**FIGURE 6:**
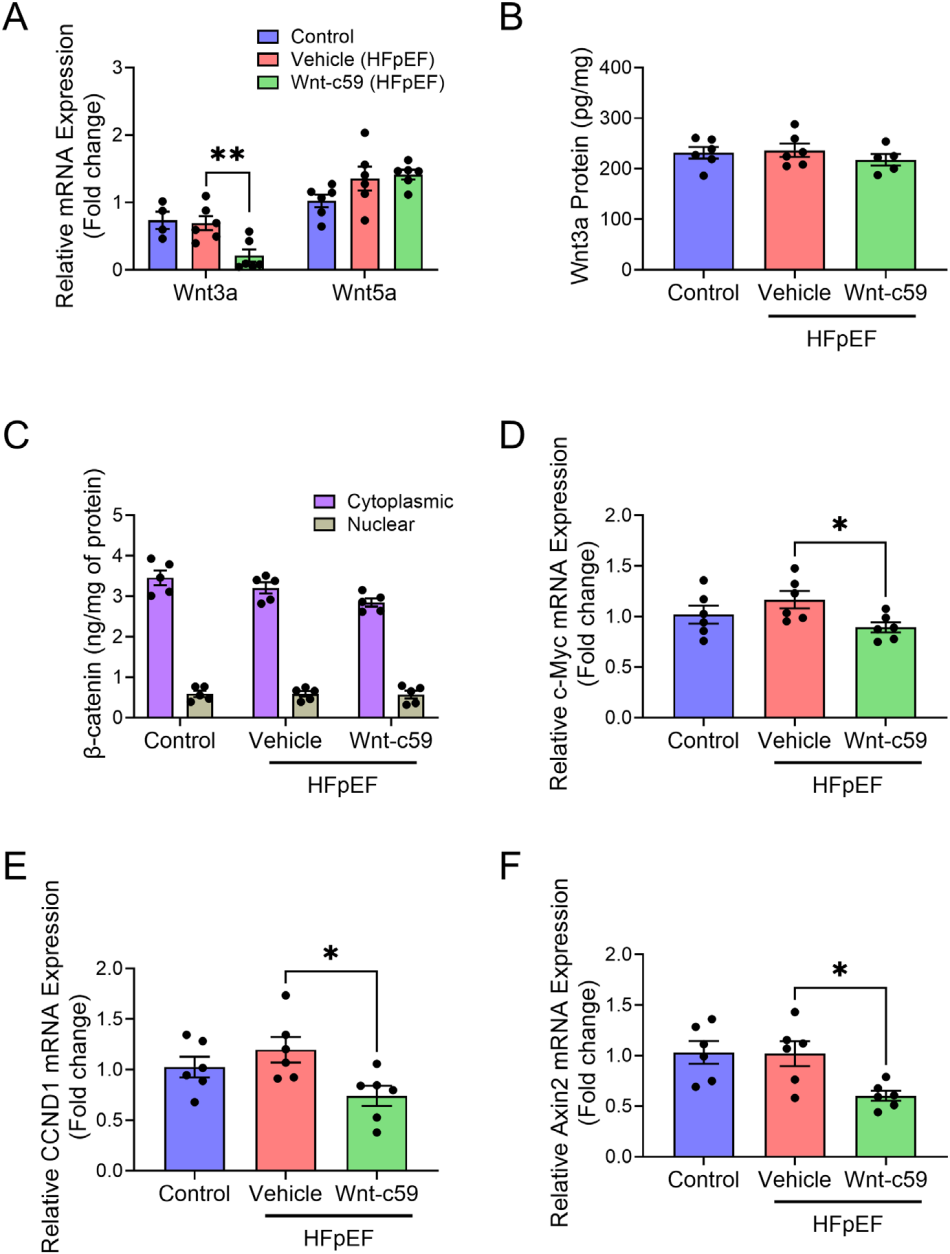
Wnt‐c59 reduced the cardiac expression of target genes of canonical Wnt signaling in HFpEF mice. Wnt3a and Wnt5a mRNA expression (A), Wnt3a protein expression (B), β‐catenin protein expression (C), c‐Myc mRNA expression (D), CCND1 mRNA expression (E), and Axin2 mRNA expression (F). Data was expressed as mean ± SEM. *p < 0.05; **p < 0.01. *n* = 5–6.

To investigate whether Wnt signaling was activated in the hearts of HFpEF mice, both the mRNA expression of Wnt ligands and target genes and the protein expression of Wnt ligands and β‐catenin were measured. Cardiac expression of both Wnt3a and Wnt5a mRNA was unaltered in the vehicle treated HFpEF mice (Figure 6A), while treatment with Wnt‐c59 significantly decreased Wnt3a mRNA expression (*p* < 0.01; Figure 6A). Conversely, the cardiac expression of the Wnt3a protein did not significantly differ between any of the experimental groups (Figure 6B). As β‐catenin accumulation in the nucleus is a universal indicator of activation of canonical Wnt signaling, both the nuclear and cytoplasmic expression of β‐catenin (non‐phosphorylated and phosphorylated) protein was measured in all samples. In further support of a lack of activation of the canonical Wnt signaling pathway, there were no changes observed in the expression of nuclear β‐catenin protein between any of the experimental groups (Figure 6C). The mRNA expression of target genes of canonical Wnt signaling (i.e., c‐Myc, CCND1, and Axin2) was also examined in the hearts of all mice. The results demonstrated that administration of a HFD and L‐NAME did not alter the mRNA expression of either c‐Myc, CCND1, or Axin2; however, treatment with Wnt‐59 significantly decreased the expression of all three genes (*p* < 0.05 for all; Figure 6D–F).

### 3.6 Wnt‐c59 Induced the Expression of BNP and Both Col1 and Col3 in Fibroblasts

**FIGURE 7:**
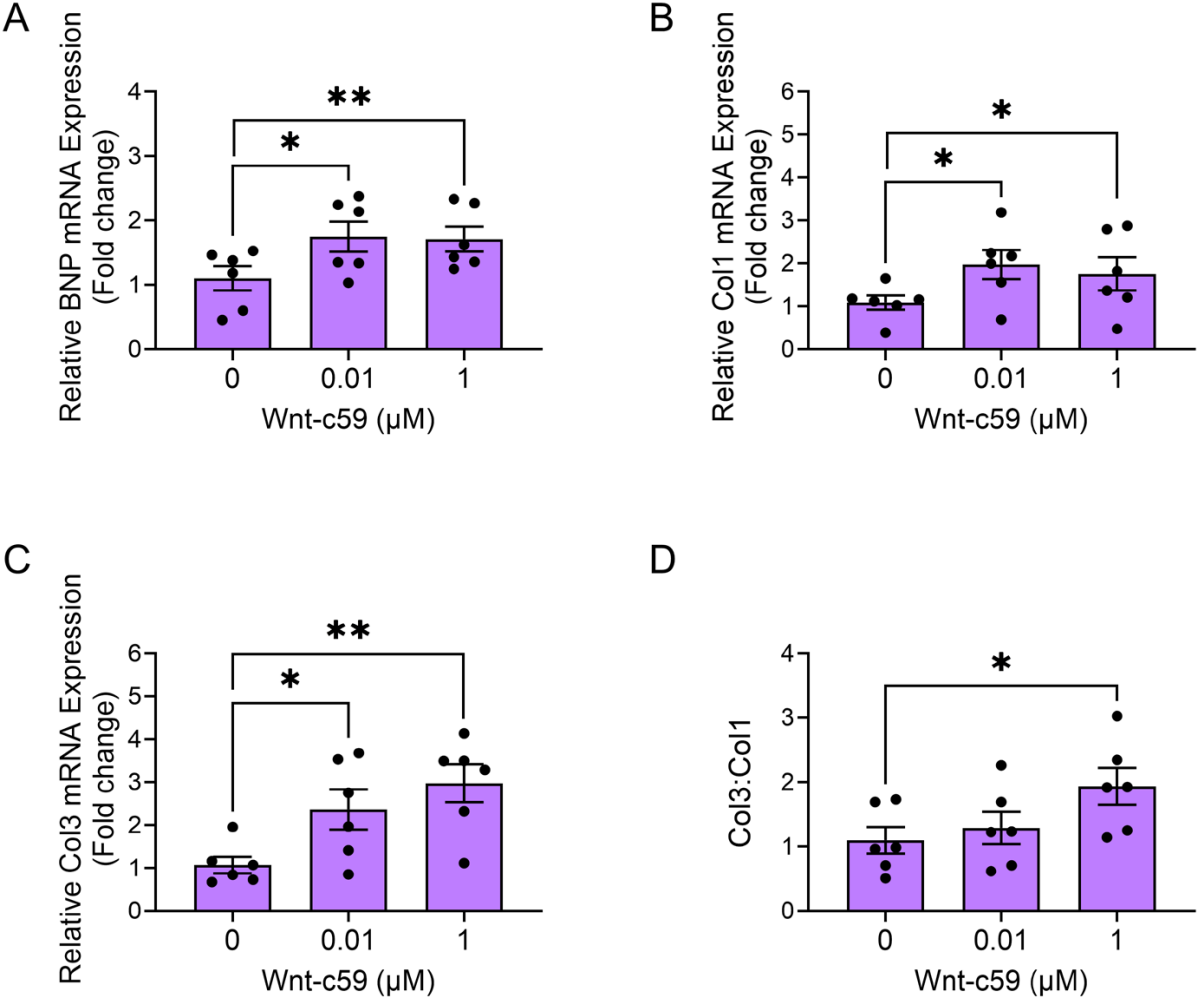
Wnt‐c59 induced the expression of both hypertrophic and fibrotic markers in mouse fibroblasts (NIH 3T3 cells). BNP (A), Col1 (B), Col3 (C), and the ratio of Col3 to Col1 (Col3:Col1) (D) mRNA expression in fibroblasts. Data was expressed as mean ± SEM. *p < 0.05; **p < 0.01. *n* = 6.

The mRNA expression of BNP was increased in mouse fibroblasts (NIH 3T3 cells) in response to both 10 nM (*p* < 0.05) and 1 μM (*p* < 0.01) Wnt‐c59 (Figure 7A). Furthermore, treatment with Wnt‐c59 also induced the mRNA expression of both Col1 (*p* < 0.05; Figure 7B) and Col3 (*p* < 0.05; Figure 7C) in fibroblasts, while the ratio of Col3:Col1 was significantly increased in response to the higher concentration of the PORCN inhibitor (*p* < 0.05; Figure 7D).

## 4 Discussion

The principle aim of this study was to investigate whether acute, oral, treatment with the PORCN inhibitor, Wnt‐c59, influenced the cardiac functional and/or structural changes induced by the “two‐hit” HFpEF model. The model was characterized by hypertension, increased adiposity, cardiac hypertrophy and fibrosis, diastolic dysfunction, and short‐term (2 weeks) administration of Wnt‐c59 improved features of both fibrosis and diastolic function. A recently published study by Wu et al. [22] demonstrated that the induction of HFpEF in experimental animals is associated with an upregulation of canonical Wnt signaling in the heart and that the cardioprotective effects (i.e., anti‐hypertrophic, anti‐fibrotic, and amelioration of diastolic dysfunction) of a different PORCN inhibitor, CGX1321, are associated with a downregulation of this signaling pathway. Conversely, in a separate study using the same HFpEF model, canonical Wnt signaling appeared to be reduced in HFpEF mice (as indicated by a reduction in β‐catenin expression), while the upregulation of this pathway seen in response to treatment with Levosimendan, a calcium‐sensitizing cardiotonic agent, was associated with improvements in diastolic function (i.e., reductions in both E/A and E/e' ratios) [33]. In the present study, canonical Wnt signaling was not activated in the hearts of HFpEF mice; however, the protective effects of Wnt‐c59 did coincide with a downregulation of mediators of canonical Wnt signaling. Taken together, this suggests that, while the cardioprotective effects of PORCN inhibitors in HFpEF are mediated in part via their modulation of Wnt signaling, other pathways and/or mechanisms may also be involved.

As anticipated, systolic function (including EF) was not altered in this experimental model of HFpEF; however, both high fat feeding and L‐NAME administration induced diastolic dysfunction (i.e., increased ventricular stiffness and impaired relaxation) as indicated by increased EDP in HFpEF mice. These findings are in agreement with those of several other studies using the same experimental model of metabolic (HFD) and mechanical (L‐NAME) stress‐induced HFpEF, which also demonstrated diastolic dysfunction (i.e., increased E/A and E/e' ratios measured via echocardiography and increased EDP and EDPVR measured via PVL) [22, 24, 26, 33, 34]. In the present study, administration of Wnt‐c59 to mice reduced the HFD and L‐NAME‐induced increase in EDP, while EDPVR values in mice treated with the PORCN inhibitor were similar to those observed in control animals. Taken together, these findings indicate that Wnt‐c59 has the potential to improve diastolic function in HFpEF mice.

One possible mechanism mediating the Wnt‐c59‐induced reduction in EDP and improved diastolic function in HFpEF mice may involve the phosphorylation of the myofilament protein, titin. Previous work has demonstrated that a reduction in protein kinase G (PKG) activity results in hypophosphorylation of titin, which consequently increases cardiomyocyte passive tension and attenuates left ventricular capacitance. PKG‐related hypophosphorylation of titin has been suggested as a key contributor to diastolic dysfunction in HFpEF [35, 36] on the basis that both cyclic guanosine monophosphate (cGMP) and PKG activity have been shown to be reduced in myocardial biopsies from patients with HFpEF [37]. Furthermore, reduced nitric oxide (NO) bioavailability has been shown to contribute to both decreased cGMP and PKG activity in clinical and experimental HFpEF [38]. In the present study, the nitric oxide synthase (NOS) inhibitor, L‐NAME, was used as the “mechanical stressor” to induce the “two‐hit” experimental of HFpEF, which has been shown elsewhere to significantly reduce NO bioavailability and consequently reduce both cGMP and PKG activity in the heart [39, 40]. It is therefore likely that NO/cGMP/PKG signaling and subsequent phosphorylation of titin is impeded in the hearts of the HFpEF mice used in this study, and this contributes to the diastolic dysfunction observed in these animals, which is worthy of further study. However, while Wnt‐c59 is unlikely to increase NO bioavailability in the face of generalized NOS inhibition by L‐NAME, it could increase both cGMP and PKG activity via its marked upregulation of the expression of BNP, a known positive regulator of cGMP/PKG signaling [41, 42]. The present study is the first to demonstrate the induction of BNP expression in the heart by a PORCN inhibitor and so the exact mechanism via which Wnt‐c59 exerts this effect has yet to be fully investigated.

The Wnt‐c59‐induced increase in BNP expression may also further contribute to the improvement in diastolic function by regulating the distribution of the different types of collagen fibers in the myocardium. The relative contributions of the different collagen types (i.e., Col1 and Col3) to the overall collagen disposition in cardiac interstitial tissue are known to influence both systolic and diastolic function. Furthermore, as adverse left ventricular remodeling progresses, the expression of the more compliant Col3 (which promotes elasticity) is decreased and replaced with the more tensile Col1, leading to stiffer ventricles and impaired diastolic function in particular [43, 44, 45]. We found that collagen deposition was significantly elevated in the hearts of HFpEF mice and that this was accompanied by a concomitant increase in both Col1 and Col3 expression. Although Wnt‐c59 did not reduce overall collagen deposition (as determined by picrosirius red staining) in the hearts of HFpEF mice, it did increase the cardiac expression of Col3 and consequently the ratio of Col3 to Col1 expression, an effect that would increase ventricular compliance and may, at least in part, explain the improved diastolic function observed in these animals. Indeed, previous studies have shown that stretch‐induced BNP expression increases Col3 but not Col1 expression in human cardiac fibroblasts [46]. Thus, the increased Col3 expression observed in the Wnt‐c59 treated mice in the present study may be mediated by increased BNP expression, possibly through an autocrine mechanism within the fibroblast. This hypothesis is supported, in part, by our in vitro data demonstrating that treatment of murine fibroblasts (NIH 3T3 cells) with Wnt‐c59 increased the expression of both BNP and the collagen types, Col1 and Col3, with the ratio of the more compliant Col3 to the more rigid Col1 also being increased.

## 5 Conclusion

This is the first study to investigate the acute therapeutic potential of a PORCN inhibitor (Wnt‐c59) in a model of HFpEF. The results demonstrated that in this “two‐hit” in vivo model, systolic function was not altered; however, diastolic dysfunction was evident as a consequence of increased ventricular stiffness. Since we did not demonstrate a role for canonical Wnt signaling in the development of HFpEF, the amelioration of diastolic dysfunction by Wnt‐c59 in our study is likely through other mechanisms, such as BNP‐induced phosphorylation of titin and/or favorable regulation of the ratio of collagen subtype expression, although this requires further investigation.

## Author Contributions

Conceptualization: Sarah K. Walsh, Emma E. Hector, Stephen J. Leslie, Erik Ryberg and Cherry L. Wainwright. Data acquisition: Mhairi A. Paul and Sarah K. Walsh. Formal analysis: Mhairi A. Paul and Sarah K. Walsh. Writing – Original draft: Mhairi A. Paul. Writing – Review and Editing: Sarah K. Walsh, Emma E. Hector, Stephen J. Leslie, Erik Ryberg and Cherry L. Wainwright. Funding acquisition: Sarah K. Walsh and Erik Ryberg.

## Ethics Statement

All animal studies were conducted under an appropriate Project License in accordance with the UK Animals (Scientific Procedures) Act 1986.

## Conflicts of Interest

The authors declare no conflicts of interest.

## Data Availability

The data that support the findings of this study are available from the corresponding author upon reasonable request.
